# Incidence of Guillain-Barré Syndrome After COVID-19 Vaccination in the Vaccine Safety Datalink

**DOI:** 10.1001/jamanetworkopen.2022.8879

**Published:** 2022-04-26

**Authors:** Kayla E. Hanson, Kristin Goddard, Ned Lewis, Bruce Fireman, Tanya R. Myers, Nandini Bakshi, Eric Weintraub, James G. Donahue, Jennifer C. Nelson, Stan Xu, Jason M. Glanz, Joshua T. B. Williams, Jonathan D. Alpern, Nicola P. Klein

**Affiliations:** 1 Marshfield Clinic Research Institute, Marshfield, Wisconsin; 2 Kaiser Permanente Vaccine Study Center, Kaiser Permanente Northern California, Oakland, California; 3 Immunization Safety Office, Centers for Disease Control and Prevention, Atlanta, Georgia; 4 The Permanente Medical Group, Oakland, California; 5 Kaiser Permanente Washington Health Research Institute, Seattle, Washington; 6 Research and Evaluation, Kaiser Permanente Southern California, Pasadena, California; 7 Kaiser Permanente Colorado Institute for Health Research, Denver, Colorado; 8 Ambulatory Care Services, Denver Health & Hospital Authority, Denver, Colorado; 9 HealthPartners Institute, Minneapolis, Minnesota

## Abstract

**Importance:**

Postauthorization monitoring of vaccines in a large population may detect rare adverse events not identified in clinical trials such as Guillain-Barré syndrome (GBS), which has a background rate of 1 to 2 per 100 000 person-years.

**Objective:**

To describe cases and incidence of GBS following COVID-19 vaccination and assess the risk of GBS after vaccination for Ad.26.COV2.S (Janssen) and mRNA vaccines.

**Design, Setting, and Participants:**

This cohort study used surveillance data from the Vaccine Safety Datalink at 8 participating integrated health care systems in the United States. There were 10 158 003 participants aged at least 12 years. Data analysis was performed from November 2021 to February 2022.

**Exposures:**

Ad.26.COV2.S, BNT162b2 (Pfizer-BioNTech), or mRNA-1273 (Moderna) COVID-19 vaccine, including mRNA vaccine doses 1 and 2, December 13, 2020, to November 13, 2021.

**Main Outcomes and Measures:**

GBS with symptom onset in the 1 to 84 days after vaccination, confirmed by medical record review and adjudication. Descriptive characteristics of confirmed cases, GBS incidence rates during postvaccination risk intervals after each type of vaccine compared with the background rate, rate ratios (RRs) comparing GBS incidence in the 1 to 21 vs 22 to 42 days postvaccination, and RRs directly comparing risk of GBS after Ad.26.COV2.S vs mRNA vaccination, using Poisson regression adjusted for age, sex, race and ethnicity, site, and calendar day.

**Results:**

From December 13, 2020, through November 13, 2021, 15 120 073 doses of COVID-19 vaccines were administered to 7 894 989 individuals (mean [SE] age, 46.5 [0.02] years; 8 138 318 doses received [53.8%] by female individuals; 3 671 199 doses received [24.3%] by Hispanic or Latino individuals, 2 215 064 doses received [14.7%] by Asian individuals, 6 266 424 doses received [41.4%] by White individuals), including 483 053 Ad.26.COV2.S doses, 8 806 595 BNT162b2 doses, and 5 830 425 mRNA-1273 doses. Eleven cases of GBS after Ad.26.COV2.S were confirmed. The unadjusted incidence rate of GBS per 100 000 person-years in the 1 to 21 days after Ad.26.COV2.S was 32.4 (95% CI, 14.8-61.5), significantly higher than the background rate, and the adjusted RR in the 1 to 21 vs 22 to 42 days following Ad.26.COV2.S was 6.03 (95% CI, 0.79-147.79). Thirty-six cases of GBS after mRNA vaccines were confirmed. The unadjusted incidence rate per 100 000 person-years in the 1 to 21 days after mRNA vaccines was 1.3 (95% CI, 0.7-2.4) and the adjusted RR in the 1 to 21 vs 22 to 42 days following mRNA vaccines was 0.56 (95% CI, 0.21-1.48). In a head-to-head comparison of Ad.26.COV2.S vs mRNA vaccines, the adjusted RR was 20.56 (95% CI, 6.94-64.66).

**Conclusions and Relevance:**

In this cohort study of COVID-19 vaccines, the incidence of GBS was elevated after receiving the Ad.26.COV2.S vaccine. Surveillance is ongoing.

## Key Points

**Question:**

Are COVID-19 vaccines associated with Guillain-Barré syndrome (GBS)?

**Findings:**

In this cohort study of surveillance data from the Vaccine Safety Datalink that included 15.1 million doses of COVID-19 vaccines, the unadjusted incidence rate of confirmed GBS in the 1 to 21 days after receiving the Ad.26.COV2.S (Janssen) vaccine was 32.4 per 100 000 person-years, which was significantly higher than the background rate of GBS. The unadjusted incidence rate of confirmed GBS in the 1 to 21 days after mRNA vaccines was 1.3 per 100 000 person-years, which did not differ from the background rate.

**Meaning:**

These findings suggest an increased risk of GBS after Ad.26.COV2.S vaccination.

## Teaser

This cohort study investigates cases and incidence of Guillain-Barré syndrome following COVID-19 vaccination and assesses the risk of GBS after vaccination with Ad.26.COV2.S (Janssen) vaccines vs mRNA vaccines.

## Introduction

Three vaccine products are available in the US to prevent COVID-19, including BNT162b2 (Pfizer-BioNTech), mRNA-1273 (Moderna), and Ad.26.COV2.S (Janssen).^1,2,3^ BNT162b2 and mRNA-1273 are both messenger RNA (mRNA) vaccines and administered as 2-dose primary series, whereas Ad.26.COV2.S is a replication-incompetent adenoviral vector vaccine and administered as a single dose primary series. mRNA vaccines were authorized for use in adults in December 2020, and use of BNT162b2 was expanded to adolescents in May 2021. Ad.26.COV2.S was authorized for use in adults on February 27, 2021; however, use of Ad.26.COV2.S was temporarily paused in mid-April 2021 due to concerns about a rare condition called thrombosis with thrombocytopenia syndrome (TTS) that was reported after Ad.26.COV2.S vaccination.^4^ In July 2021, data from the Vaccine Adverse Event Reporting System (VAERS) indicated that the reporting rate of Guillain-Barré syndrome (GBS), a rare neurological disorder, was higher after Ad.26.COV2.S than after mRNA vaccines.^5^ The Food and Drug Administration subsequently added a warning about GBS to the Ad.26.COV2.S vaccine fact sheet. On December 16, 2021, the Advisory Committee on Immunization Practices made a preferential recommendation for use of mRNA vaccines over Ad.26.COV2.S as the benefit-risk balance was determined to be more favorable for mRNA vaccines than Ad.26.COV2.S, in part due to safety concerns regarding GBS and TTS after Ad.26.COV2.S.^6^

Postauthorization monitoring of vaccines in a large population can detect rare adverse events not identified in clinical trials. GBS is being monitored in the Vaccine Safety Datalink as part of ongoing rapid and prospective COVID-19 vaccine safety surveillance efforts.^7^ Our objectives for this study were to (1) describe GBS cases and incidence following COVID-19 vaccinations from December 13, 2020, through November 13, 2021, and (2) assess the risk of GBS after vaccination for Ad.26.COV2.S and mRNA vaccines.

## Methods

### Setting and Population

The Vaccine Safety Datalink is a collaboration between 9 US integrated health care systems and the Centers for Disease Control and Prevention (CDC).^8,9^ Eight data-contributing organizations (Kaiser Permanente: Colorado, Northern California, Northwest, Southern California, and Washington; Marshfield Clinic; HealthPartners; and Denver Health) have access to comprehensive medical records, including vaccinations, for 10 158 003 people aged at least 12 years as of November 10, 2021.

### Study Design

This activity was approved by the institutional review boards with a waiver of informed consent at Kaiser Permanente: Colorado, Northern California, Northwest, Southern California, and Washington; Marshfield Clinic; HealthPartners; and Denver Health. Per 45 CFR part 46.111(f)(3), this study was granted a waiver of informed consent because it posed minimal risk to participants and could not feasibly be conducted without the waiver. The CDC determined that this activity was public health surveillance (45 CFR part 46.102[l][2]) and thus did not require institutional review board review. This report followed the Strengthening the Reporting of Observational Studies in Epidemiology (STROBE) reporting guideline.^10^

Since December 2020, the Vaccine Safety Datalink has been conducting safety surveillance of COVID-19 vaccines, monitoring GBS and 22 other prespecified, serious outcomes after COVID-19 vaccines on a weekly basis. Methods are described in the study protocol and in a prior publication that includes interim results for mRNA vaccines.^7,11^ Weekly analyses compared outcome incidence observed during a risk interval after vaccination (eg, 1-21 days after Ad.26.COV2.S) with outcome incidence expected. Analyses were conducted separately by vaccine type. The expected was derived from 1 of 2 types of comparators: (1) similar vaccinated persons who were concurrently (on the same calendar day) in a postvaccination comparison interval following the same vaccine type (eg, 22-42 days after Ad.26.COV2.S) (vaccinated concurrent comparators), and (2) similar persons who were concurrently (on the same calendar day) unvaccinated (ie, had not received any doses of COVID-19 vaccine) (unvaccinated concurrent comparators), where similar means comparators were of the same age group, sex, race or ethnicity, and site as the vaccine recipient. Race and ethnicity data were captured in Vaccine Safety Datalink files in fixed categories based on self-reported data from the participating sites. Race and ethnicity data were used to adjust for confounding that may have arisen if the factor was associated with vaccination dates and outcome events. By protocol, vaccinated concurrent comparators were considered primary and unvaccinated concurrent comparators were considered supplemental owing to concerns about possible bias from unmeasured differences between vaccinated and unvaccinated individuals. This surveillance approach used 2 postvaccination risk intervals, 1 to 21 days and 1 to 42 days, with corresponding postvaccination comparison intervals for vaccinated concurrent comparator analyses, 22 to 42 days and 43 to 84 days, respectively. The 1 to 21–day risk interval allowed for timelier analyses and avoids bias introduced from the short interval between doses for mRNA vaccines. However, a 1 to 42–day risk interval was also used, because this interval is often used in vaccine safety studies of GBS and other outcomes.^12^ Individuals who received 2 doses of an mRNA vaccine contributed to analyses of mRNA vaccines when they were 1 to 21 (or 1 to 42) days after dose 1 and again when they were 1 to 21 (or 1 to 42) days after dose 2. However, receipt of dose 2 resulted in censoring of follow-up time after dose 1, therefore most comparison time in the vaccinated concurrent comparator analyses was after dose 2.

We reported on data through November 13, 2021, including descriptive characteristics of COVID-19 vaccine recipients and GBS cases, weekly analyses of GBS with vaccinated and unvaccinated concurrent comparators (previously described) for Ad.26.COV2.S and mRNA vaccines, and additional GBS analyses including comparisons of the incidence of GBS postvaccination by vaccine type to the incidence expected from prior studies, evaluation of the temporal clustering of GBS cases following Ad.26.COV2.S, and head-to-head comparisons of GBS incidence after Ad.26.COV2.S with GBS incidence after mRNA vaccination.

Potential cases of GBS were identified among Vaccine Safety Datalink members aged at least 12 years using *International Statistical Classification of Diseases and Related Health Problems, Tenth Revision (ICD-10)* code G61.0 in the emergency department or inpatient setting, specifically when G61.0 first appeared in an individual’s record in the 1 to 84 days after dose 1 or dose 2 of an mRNA vaccine or after dose 1 of Ad.26.COV2.S. Because disease onset may begin before a diagnosis is recorded in the medical record, potential cases with the *ICD-10* code in the 85 to 98 days postvaccination were also reviewed. After review, all cases underwent adjudication according to the Brighton Collaboration criteria.^13^ Briefly, GBS cases meeting Brighton level 1 had the highest level of diagnostic certainty, whereas level 4 included cases of suspected GBS (ie, insufficient information was available in the medical record to meet Brighton level 1-3). In this analysis, we considered Brighton level 1 to level 4 cases as confirmed and sensitivity analyses were conducted excluding level 4 cases. Cases of GBS among unvaccinated concurrent comparators were identified by applying the same algorithm to unvaccinated members of the Vaccine Safety Datalink population during the period of interest. Unvaccinated comparators did not undergo medical record review and adjudication.

### Statistical Analysis

As previously described,^7^ primary weekly monitoring of GBS after each type of COVID-19 vaccine used conditional Poisson regression to compare individuals during a postvaccination risk interval with similar individuals who had been vaccinated earlier and were concurrently in a comparison interval. These analyses estimated rate ratios (ie, ratios of incidence rates in a risk interval divided by incidence rates in a comparison interval) that were adjusted for 5-year age group, sex, race and ethnicity, site, and calendar day by conditioning the Poisson regression on strata (risk sets) defined by these factors. Similarly, conditional Poisson regression was used in supplementary weekly analyses to compare individuals in a postvaccination risk interval with similar individuals who were concurrently unvaccinated. One-sided sequential testing was conducted weekly for vaccinated concurrent comparator analyses, with a signaling threshold of *P* < .0048 (prespecified to keep the overall chance of making a type I error below .05 during 2 years of weekly analyses).

Although our primary weekly analyses of GBS have not met the criterion for a safety signal, concerns about GBS after Ad.26.COV2.S arose from supplementary analyses with unvaccinated comparators and from reports to VAERS. To further investigate we conducted additional analyses. We computed unadjusted incidence rates and 95% CIs of confirmed GBS in the 1 to 21 days and 1 to 42 days following COVID-19 vaccination per 100 000 person-years (PY) by vaccine type and used exact Poisson regression to compare observed incidence rates to the historical background rate of GBS (2 per 100 000 PY).^14,15^ We also examined the temporal clustering of the GBS cases within 56 days of Ad.26.COV2.S by day of symptom onset, using a scan statistic.^16^ Lastly, we conducted head-to-head comparisons of the 21-day and 42-day risk intervals after Ad.26.COV2.S vs the 21-day and 42-day risk intervals after mRNA vaccination, using conditional Poisson regression to estimate rate ratios (ie, the ratio of GBS incidence after Ad.26.COV2.S divided by GBS incidence after mRNA vaccination), adjusted for age group, sex, race and ethnicity, site, and calendar day as described above. All analyses of mRNA vaccines combined both products (BNT162b2 and mRNA-1273) and primary series doses (1 and 2) to increase statistical power. Analyses were 2-sided with a .05 level of significance unless otherwise specified and were conducted using SAS version 9.4 (SAS Institute) from November 2021 to February 2022.

## Results

### COVID-19 Vaccinations

From December 13, 2020, through November 13, 2021, 15 120 073 COVID-19 vaccines were administered to 7 894 989 individuals who were at least 12 years of age (mean age [SE] age, 46.5 [0.02] years; 8 138 318 doses received [53.8%] by female individuals; 3 671 199 doses received [24.3%] by Hispanic or Latino individuals, 2 215 064 doses received [14.7%] by Asian individuals, 6 266 424 doses received [41.4%] by White individuals), including 483 053 first doses of Ad.26.COV2.S, 8 806 595 first and second doses of BNT162b2, and 5 830 425 first and second doses of mRNA-1273; mRNA vaccines accounted for 96.8% of all doses (14 637 020 of 15 120 073). Compared with mRNA vaccine recipients, a higher proportion of Ad.26.COV2.S vaccine recipients were male (6 721 529 [45.9%] vs 260 226 [53.9%]) and a smaller proportion were at least 65 years of age (3 139 195 [21.5%] vs 55 901 [11.6%]) (Table 1). Ad.26.COV2.S vaccination was concentrated during the 7-week period that corresponded with the period between initial authorization and the pause in use due to concerns about TTS, with 63.5% of Ad.26.COV2.S doses (306 590 of 483 053) administered from February 28, 2021, through April 17, 2021.

**Table 1.** COVID-19 Vaccine Doses According to Characteristics of the Vaccine Recipient

Characteristic	Vaccine doses, No. (%) (N = 15 120 073)^a^	
	Ad.26.COV2.S (n = 483 053)	mRNA vaccine (n = 14 637 020)^b^
Dose number		
1	483 053 (100.0)	7 411 936 (50.6)
2	NA	7 225 084 (49.4)
Sex		
Male	260 226 (53.9)	6 721 529 (45.9)
Female	222 827 (46.1)	7 915 491 (54.1)
Age group, y		
12-17	NA	1 132 109 (7.7)
18-49	260 540 (53.9)	6 874 592 (47.0)
50-64	166 612 (34.5)	3 491 124 (23.9)
65-74	37 567 (7.8)	1 917 458 (13.1)
≥75	18 334 (3.8)	1 221 737 (8.4)
Race and ethnicity^c^		
Hispanic or Latino	101 804 (21.1)	3 569 395 (24.4)
Non-Hispanic		
American Indian or Alaska Native	1600 (0.3)	43 984 (0.3)
Asian	55 908 (11.6)	2 159 156 (14.8)
Black	31 978 (6.6)	885 540 (6.1)
Native Hawaiian or Pacific Islander	2805 (0.6)	93 050 (0.6)
White	221 468 (45.9)	6 044 956 (41.3)
Multiple/other	14 975 (3.1)	510 068 (3.5)
Unknown	52 515 (10.9)	1 330 871 (9.1)
History of COVID-19	43 183 (8.9)	1 091 912 (7.5)

Abbreviation: NA, not applicable.

^a^ Percentages may not add to 100% due to rounding.

^b^ Includes 8 806 595 doses of BNT162b2 and 5 830 425 doses of mRNA-1273.

^c^ Eight combined race and ethnicity categories were created from available race and ethnicity information. Persons were classified as Hispanic or Latino if categorized as having Hispanic or Latino ethnicity, regardless of whether or not race was known. Persons with more than 1 non-Hispanic race or ethnicity and all other non-Hispanic races and ethnicities were classified as Multiple/Other. Persons were classified as having unknown race and ethnicity if both race and ethnicity were missing.

### GBS After Ad.26.COV2.S

During the 1 to 84 days after individuals received Ad.26.COV2.S, 22 potential cases of GBS were identified: all were reviewed and adjudicated, and 11 (50%) were confirmed. Symptom onset ranged from 1 to 77 days after Ad.26.COV2.S; 9 cases (82%) had symptom onset in the 1 to 21 days postvaccination, 1 (9%) had symptom onset in the 22 to 42 days postvaccination, and 1 (9%) had symptom onset in the 43 to 84 days postvaccination (Figure A). Scan statistics identified days 1 to 14 after vaccination as a statistically significant cluster (*P* = .003). Ten patients were hospitalized (91%) and all were treated with intravenous immune globulin. Patients with confirmed GBS after Ad.26.COV2.S had a mean (range) age of 50 (32-63) years, 9 (82%) were male, 10 (91%) were non-Hispanic White, and 10 (91%) had facial weakness or paralysis in addition to bilateral weakness or paralysis of the limbs (Table 2).

**Figure.**
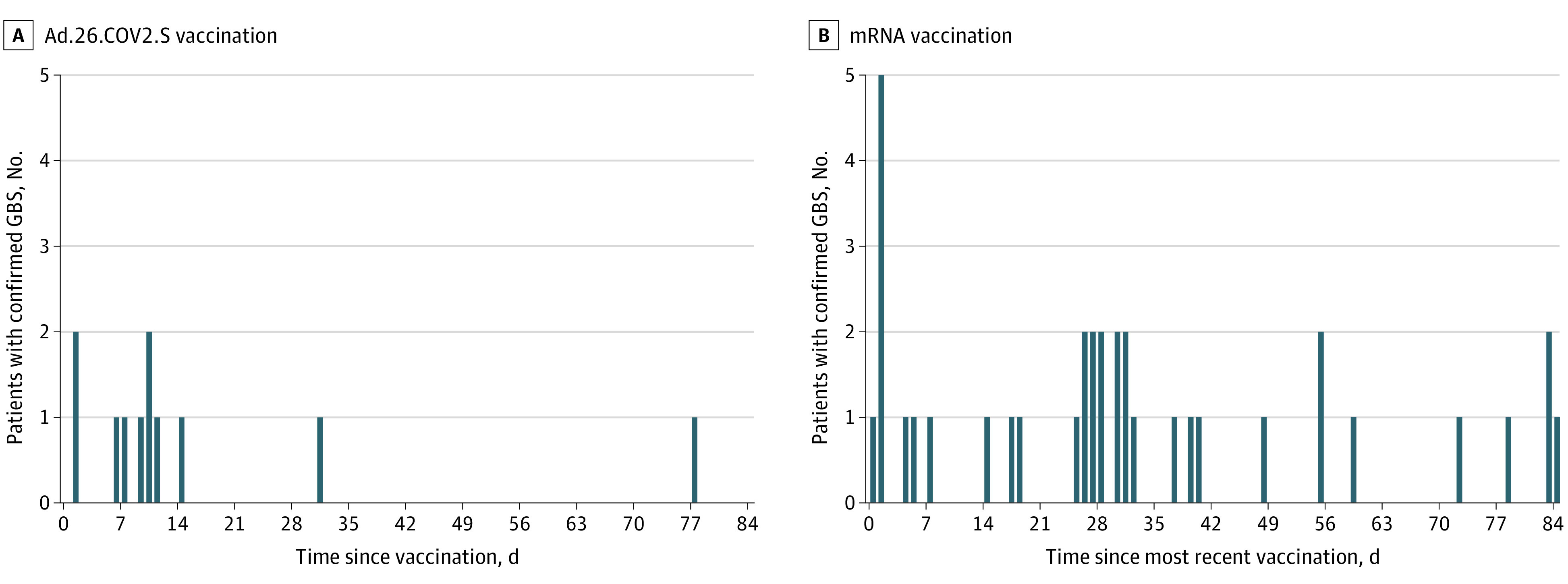
Timing of GBS Symptom Onset after COVID-19 Vaccination A, Bars represent the number of confirmed cases of GBS after Ad.26.COV2.S vaccination (N = 11). The x-axis denotes the numbers of days between Ad.26.COV2.S vaccination and GBS symptom onset. The 84-day follow-up period has elapsed for all Ad.26.COV2.S vaccinations. A statistically significant cluster was identified from days 1 to 14 after Ad.26.COV2.S vaccination using scan statistics (*P* = .003).^16^ B, Bars represent the number of confirmed cases of GBS after mRNA vaccination (N = 36). The x-axis denotes the numbers of days between most recent mRNA vaccination and GBS symptom onset. The 84-day follow-up period has elapsed for all mRNA vaccinations.

**Table 2.** Characteristics of Confirmed GBS Cases After COVID-19 Vaccination

Characteristic	Cases, No. (%)^a^			
	All confirmed GBS cases^b^		Confirmed GBS cases with symptom onset in the 1-21 d after vaccination	
	Ad.26.COV2.S (n = 11)	mRNA vaccine (n = 36)	Ad.26.COV2.S (n = 9)	mRNA vaccine (n = 11)
Age, mean (SD), y	50 (12)	53 (20)	54 (9)	63 (18)
Age group, y				
12-17	NA	2 (6)	NA	0
18-49	4 (36)	15 (42)	2 (22)	4 (36)
50-64	7 (64)	5 (14)	7 (78)	1 (9)
≥65	0	14 (39)	0	6 (55)
Sex				
Male	9 (82)	18 (50)	7 (78)	5 (45)
Female	2 (18)	18 (50)	2 (22)	6 (55)
Race and ethnicity				
Hispanic or Latino	1 (9)	12 (33)	1 (11)	2 (18)
White, non-Hispanic	10 (91)	15 (42)	8 (89)	6 (55)
Other, non-Hispanic or unknown^c^	0	9 (25)	0	3 (27)
History of COVID-19	0	6 (17)	0	0
Recent receipt of other vaccine(s)^d^	0	1 (3)	0	0
Recent respiratory or gastrointestinal illness^e^	2 (18)	8 (22)	1 (11)	0
No. of COVID-19 vaccine doses received prior to symptom onset				
1	11 (100)	13 (36)	9 (100)	8 (73)
2	NA	23 (64)	NA	3 (27)
mRNA vaccine product				
BNT162b2	NA	23 (64)	NA	6 (55)
mRNA-1273	NA	13 (36)	NA	5 (45)
Days to symptom onset from most recent COVID-19 vaccine, median (IQR)	10 (6-14)	28 (11-44)	9 (6-10)	4 (1-14)
Symptoms				
Bilateral flaccid weakness or paralysis of the limbs	11 (100)	35 (97)	9 (100)	11 (100)
Decreased or absent deep tendon reflexes in the limbs	10 (91)	33 (92)	8 (89)	10 (91)
Cranial nerve/facial muscle paralysis or weakness	10 (91)	12 (33)	8 (89)	4 (36)
Extraocular muscle paralysis or weakness	1 (9)	4 (11)	1 (11)	0
Ataxia	6 (55)	22 (61)	4 (44)	6 (55)
Elevated protein in the CSF^f^^,^^g^	8 (80) [10]	23 (77) [30]	7 (88) [8]	8 (89) [9]
Elevated white blood cells in the CSF^f^^,^^h^	1 (11) [9]	0 [26]	1 (13) [8]	0 [8]
Abnormal EMG or nerve conduction study^f^	2 (100) [2]	12 (100) [12]	2 (100) [2]	3 (100) [3]
Hospital length of stay, median (IQR), d^i^	5 (4-13) [10]	6 (5-10)	5 (4-15) [8]	5 (4-6)
Admitted to the ICU	1 (9)	4 (11)	1 (11)	1 (9)
Treatment				
IVIG	11 (100)	35 (97)	9 (100)	11 (100)
Corticosteroids	2 (18)	9 (25)	2 (22)	1 (9)
Plasmapheresis	3 (27)	4 (11)	3 (33)	2 (18)
Intubation and mechanical ventilation	1 (9)	4 (11)	1 (11)	1 (9)
Outcome^j^				
Deceased^k^	0	2 (6)	0	1 (9)
Recovered with no neurologic sequelae	0	4 (11)	0	1 (9)
Illness ongoing	11 (100)	30 (83)	9 (100)	9 (82)
Brighton level^l^				
1	1 (9)	3 (8)	1 (11)	0
2	7 (64)	23 (64)	6 (67)	7 (64)
3	2 (18)	5 (14)	1 (11)	2 (18)
4	1 (9)	5 (14)	1 (11)	2 (18)

Abbreviations: CSF, cerebrospinal fluid; EMG, electromyography; GBS, Guillain-Barré syndrome; ICU, intensive care unit; IVIG, intravenous immune globulin.

^a^ Percentages may not add to 100% due to rounding.

^b^ 53 cases were not confirmed after medical record review and adjudication, including 11 cases after Ad.26.COV2.S and 42 cases after mRNA vaccination. Reasons included history of GBS with no new or worsening symptoms (n = 19), GBS ruled out (n = 13), case did not meet Brighton criteria for GBS or Miller-Fisher syndrome (Brighton Level 5) (n = 12), no evidence that GBS was diagnosed on or around the automated diagnosis date in the medical record (n = 4), GBS symptom onset prior to COVID-19 vaccination (n = 3), history of chronic inflammatory demyelinating polyneuropathy (CIDP) with no new or worsening symptoms (n = 1), and indeterminate symptom onset (n = 1).

^c^ Race and ethnicity categories were collapsed owing to the small number of cases observed. Other/unknown includes all persons with non-Hispanic ethnicity and from minoritized racial and ethnic groups, as well as those who identified as multiracial or had unknown race and ethnicity.

^d^ Medical records were reviewed to determine if the patient received any non-COVID-19 vaccines in the 2 months prior to GBS diagnosis.

^e^ Medical records were reviewed to determine if the patient had a gastrointestinal or respiratory illness (including COVID-19) in the 2 months prior to GBS symptom onset. Confirmatory laboratory testing was not required.

^f^ Test not performed for all patients; number with test performed is shown in brackets and percentage in parenthesis is calculated out of those with test performed.

^g^ Elevated protein defined as greater than laboratory reference, which is greater than 45 mg/dL at most laboratories.

^h^ Elevated white blood cells defined as greater than or equal to 50 cells/μL.

^i^ Hospital length of stay is not available for 1 case after Ad.26.COV2.S because they were treated in the emergency department and not admitted to the hospital.

^j^ Outcome was assessed at the time of medical record review, which was typically around 30 days after their initial diagnosis.

^k^ Both cases after mRNA vaccination that were noted as deceased at the time of medical record review were greater than 80 years of age and had multiple underlying health conditions. Cause of death was unknown for one individual, although they were noted to have been admitted to hospice care for bladder cancer. For the other individual cause of death was multiorgan failure with GBS listed as one of several contributory factors.

^l^ Cases were adjudicated according to the Brighton Collaboration criteria.^13^ Briefly, levels 1 to 3 meet the Brighton case definition for GBS, with level 1 the highest level of diagnostic certainty, and level 4 are suspected cases (diagnosed as GBS but insufficient evidence to meet the Brighton case definition). Cases adjudicated as Brighton levels 1 to 4 were considered confirmed.

The unadjusted incidence rate of confirmed cases of GBS per 100 000 PY was 32.4 (95% CI, 14.8-61.5) during the 1 to 21 days after Ad.26.COV2.S and 18.0 (95% CI, 8.6-33.1) during the 1 to 42 days after Ad.26.COV2.S; both estimates were significantly higher than the background rate of GBS (*P* < .001) (Table 3). When excluding Brighton level 4 cases, incidence rates were lower (28.8 [95% CI, 12.4-56.8] and 16.2 [95% CI, 7.4-30.8] per 100 000 PY, respectively) but still significantly higher than the background rate (*P* < .001).

**Table 3.** Incidence Rate of Confirmed GBS in the 1 to 21 Days and 1 to 42 Days After COVID-19 Vaccination

Vaccine type	Risk window, d	Including BL 4 cases^a^	No.			Unadjusted incidence rate (95% CI)		*P* value, 2-sided^c^
			GBS cases	Vaccine doses	Person-years^b^	Per million doses	Per 100 000 person-years	
Ad.26.COV2.S	1-21	Yes	9	483 053	27 773	18.6 (8.5-35.4)	32.4 (14.8-61.5)	<.001
		No	8	483 053	27 773	16.6 (7.2-32.6)	28.8 (12.4-56.8)	<.001
	1-42	Yes	10	483 053	55 546	20.7 (9.9-38.1)	18.0 (8.6-33.1)	<.001
		No	9	483 053	55 546	18.6 (8.5-35.4)	16.2 (7.4-30.8)	<.001
mRNA vaccine	1-21	Yes	11	14 637 020	831 790	0.8 (0.4-1.3)	1.3 (0.7-2.4)	.20
		No	9	14 637 020	831 790	0.6 (0.3-1.2)	1.1 (0.5-2.1)	.06
	1-42	Yes	26	14 637 020	1 329 815	1.8 (1.2-2.6)	2.0 (1.3-2.9)	.99
		No	23	14 637 020	1 329 815	1.6 (1.0-2.4)	1.7 (1.1-2.6)	.56

Abbreviations: BL, Brighton level; GBS, Guillain-Barré syndrome.

^a^ Sensitivity analyses were conducted excluding Brighton level 4 cases (suspected cases).

^b^ Follow-up time after dose 1 of either mRNA vaccine was censored after receipt of dose 2.

^c^ The background rate of GBS is 1 to 2 per 100 000 person-years.^14,15^ Exact Poisson regression was used to compare the observed number of GBS cases with the expected number of cases, which was derived from a background rate of 2 per 100 000 person-years and the observed number of person-years.

In weekly surveillance using vaccinated concurrent comparators, as of November 16, 2021, the RR of confirmed GBS, adjusted for age, sex, race and ethnicity, site, and calendar day in the 1 to 21 vs 22 to 42 days following Ad.26.COV2.S was 6.03 (95% CI, 0.79-147.79; 2-sided *P* = .09; 1-sided *P* = .08). The adjusted RR of confirmed GBS in the 1 to 42 vs 43 to 84 days following Ad.26.COV2.S was 8.64 (95% CI, 1.18-207.32; 2-sided *P* = .03; 1-sided *P* = .03). Neither result met the prespecified signaling criteria of a 1-sided *P* < .0048. In supplemental weekly analyses with unvaccinated concurrent comparators, the adjusted RR of GBS was 10.57 (95% CI, 5.15-20.16; *P* < .001) in the 1 to 21 days following Ad.26.COV2.S and 10.05 (95% CI, 5.75-16.96; *P* < .001) in the 1 to 42 days following Ad.26.COV2.S.

### GBS After mRNA Vaccines

During the 1 to 84 days after first and second doses of mRNA vaccines, 78 potential cases of GBS were identified: all were reviewed and adjudicated, and 36 (46%) were confirmed, including 23 after BNT162b2 and 13 after mRNA-1273. Patients with confirmed GBS after mRNA vaccines had a mean (range) age of 53 (14-92) years (Table 2) and symptom onset ranged from 0 to 84 days after vaccination (Figure B). Eleven cases (31%) had symptom onset in the 1 to 21 days postvaccination, 15 cases (42%) had symptom onset in the 22 to 42 days postvaccination, and 9 cases (25%) had symptom onset in the 43 to 84 days postvaccination. One case had symptom onset on the same day as mRNA vaccination (day 0) and was excluded from analyses.

The unadjusted incidence rate of confirmed cases of GBS per 100 000 PY in the 21 days after mRNA vaccines was 1.3 (95% CI, 0.7-2.4) and did not differ from the background rate of GBS (*P* = .20) (Table 3). Results were similar when excluding Brighton level 4 cases and when using a 42-day risk window, with incidence rates ranging from 1.1 to 2.0 cases of GBS per 100 000 PY.

In weekly surveillance conducted on November 16, 2021, the adjusted RR of confirmed GBS in the 1 to 21 vs 22 to 42 days following mRNA vaccination was 0.56 (95% CI, 0.21-1.48; 2-sided *P* = .25, 1-sided *P* = .93). In supplemental weekly analyses with unvaccinated concurrent comparators, the adjusted RR of GBS was 0.83 (95% CI, 0.50-1.33; *P* = .45) in the 1 to 21 days following mRNA vaccination and 0.85 (95% CI, 0.57-1.27; *P* = .44) in the 1 to 42 days following mRNA vaccination.

### Head-to-Head Comparisons

During the 1 to 21 days after vaccination, the adjusted RR of confirmed GBS after Ad.26.COV2.S vs mRNA vaccination was 20.56 (95% CI, 6.94-64.66; *P* < .001), with 15.5 excess GBS cases in the risk interval per million Ad.26.COV2.S vaccine recipients (Table 4). When using a 1 to 42-day risk interval, the adjusted RR was lower, 11.46 (95% CI, 4.83-26.16; 2-sided *P* < .001), otherwise results were similar.

**Table 4.** Head-to-Head Comparisons of Confirmed GBS Incidence After Ad.26.COV2.S vs mRNA Vaccination

Risk window, d	No.		Adjusted RR (95% CI)^b^	*P* value, 2-sided	Excess cases in risk interval per million doses
	GBS cases after Ad.26.COV2.S	GBS cases after mRNA vaccination^a^			
1-21	9	8	20.56 (6.94-64.66)	<.001	15.5
1-42	10	21	11.46 (4.83-26.16)	<.001	17.5

Abbreviations: GBS, Guillain-Barré syndrome; RR, rate ratio.

^a^ Not all confirmed cases of GBS after mRNA vaccination are included in this analysis, such as cases that occurred prior to the authorization of Ad.26.COV2.S.

^b^ Adjusted for 5-year age group, sex, race and ethnicity, site, and calendar day.

## Discussion

In this cohort study’s interim analyses conducted in a large population-based surveillance system that included medical record review of all potential GBS cases after COVID-19 vaccination through November 13, 2021, findings were consistent with an increased risk of GBS after Ad.26.COV2.S. The incidence of GBS in the 21 days after Ad.26.COV2.S was 32.4 per 100 000 person-years, which was substantially greater than the expected background rate of 1 to 2 per 100 000 person-years.^14,15^ GBS incidence in the 21 days after mRNA vaccination was 1.3 per 100 000 person-years, similar to the overall expected background rate. In an adjusted head-to-head comparison, GBS incidence during the 21 days after Ad.26.COV2.S was 20.6 times higher than the GBS incidence during the 21 days after mRNA vaccination, amounting to 15.5 excess cases per million Ad.26.COV2.S vaccine recipients. The majority of cases of GBS after Ad.26.COV2.S occurred during the 1 to 21-day risk interval, with the period of most increased risk in the 1 to 14 days after Ad.26.COV2.S.

None of the primary analyses conducted in routine weekly safety surveillance of GBS after Ad.26.COV2.S met the criterion for a safety signal. However, these analyses had low power not only because the uptake of Ad.26.COV2.S was low, but also because Ad.26.COV2.S uptake was concentrated in a brief calendar period, meaning that relatively few vaccinated concurrent comparators were available while vaccine recipients were in their risk interval. The rate ratio estimate in the primary analyses of the 21-day risk interval after Ad.26.COV2.S had a very wide confidence interval extending from less than 1.0 to greater than 100; the CI was narrower for the corresponding supplemental analyses, ranging from 5 to 20. Because our primary analyses for GBS after Ad.26.COV2.S were less powerful than our supplementary analyses (as evidenced by wider CIs), we gave more consideration to supplemental analyses, consistent with the study protocol. Nevertheless, results of supplemental analyses should be interpreted with caution because of concerns about unmeasured differences between vaccinated and unvaccinated individuals, and because cases of GBS in unvaccinated comparators did not undergo medical record review and adjudication.

Our finding of an elevated risk of GBS after Ad.26.COV2.S was consistent with an observed-to-expected analysis in VAERS which found that the GBS reporting rate after Ad.26.COV2.S exceeded the background rate.^17^ Our analyses also included GBS cases confirmed by medical record review and adjudication, and thus provide validation of the preliminary VAERS findings based on presumptive GBS.

Interestingly, nearly all patients with GBS after Ad.26.COV2.S identified in this surveillance had facial weakness or paralysis, in addition to weakness and decreased reflexes in the limbs. Reports describing cases of GBS following Ad.26.COV2.S and ChAdOx1 (AstraZeneca) COVID-19 vaccine, another adenoviral vector vaccine used outside of the US, have also noted a preponderance of cases with unilateral or bilateral facial weakness or plegia,^18,19,20,21^ with some suggesting that the presentation of GBS after COVID-19 adenoviral vector vaccine may be novel. However, a study in the United Kingdom did not find a significant difference in presence of unilateral or bilateral facial weakness between those with GBS onset in the 42 days after COVID-19 vaccination (majority first doses of ChAdOx1) and those with GBS onset more than 42 days after COVID-19 vaccination or those with GBS onset during the same period that were not vaccinated.^22^ More research is needed to determine if the presentation of GBS after adenoviral vector vaccine differs from GBS after other exposures such as *Campylobacter jejuni*, and to investigate the mechanism for how adenoviral vector vaccines may cause GBS.

In contrast to Ad.26.COV2.S, the unadjusted incidence rates of GBS in the 1 to 21 days and 1 to 42 days after mRNA vaccines were similar to the published background rate.^14,15^ In weekly analyses, the incidence of GBS following mRNA vaccination was also not significantly higher in the 1 to 21 days postvaccination compared with 22 to 42 days postvaccination, a finding consistent with similar results reported through June 22, 2021, from this same surveillance.^7^ These updated results provide further evidence that mRNA vaccines do not appear to be associated with GBS.

### Limitations

This study had several limitations. First, as mentioned previously, substantially fewer doses of Ad.26.COV2.S were administered relative to mRNA vaccines, resulting in reduced statistical power and wide confidence intervals for some analyses. Second, in this observational study recipients of Ad.26.COV2.S may have differed from recipients of mRNA vaccines in unknown ways that affect GBS risk but were not adjusted for in analyses. Third, the incidence rate of confirmed GBS during the COVID-19 pandemic has not been established, and may differ from prepandemic background rates. Fourth, we could not identify subgroups who may be at greatest risk for GBS after Ad.26.COV2.S given the small number of confirmed GBS cases identified in this surveillance. Fifth, this analysis only included GBS cases after primary COVID-19 vaccination and results may not be generalizable to additional or booster doses.

## Conclusions

In this interim analysis of surveillance data of COVID-19 vaccines, findings were consistent with an elevated risk of GBS after primary Ad.26.COV2.S vaccination. No difference in GBS risk was found for mRNA vaccines. Surveillance is ongoing.
